# Can ChatGPT Guide Parents on Tympanostomy Tube Insertion?

**DOI:** 10.3390/children10101634

**Published:** 2023-09-30

**Authors:** Alexander Moise, Adam Centomo-Bozzo, Ostap Orishchak, Mohammed K Alnoury, Sam J. Daniel

**Affiliations:** 1 Faculty of Medicine and Health Sciences, McGill University, Montreal, QC H3G 2M1, Canada; alexander.moise@mail.mcgill.ca; 2 Faculty of Dental Medicine and Oral Health Sciences, McGill University, Montreal, QC H3A 3E8, Canada; adam.centomo-bozzo@mail.mcgill.ca; 3 Department of Pediatric Otolaryngology, Montreal Children’s Hospital, Montreal, QC H4A 3J1, Canada; orishchak@ifnmu.edu.ua; 4 Department of Otolaryngology, Head and Neck Surgery, King Abdulaziz University, Jeddah 21589, Saudi Arabia; malnoury@kau.edu.sa

**Keywords:** ChatGPT, OpenAI, artificial intelligence, tympanostomy tube insertion, otolaryngology, myringotomy

## Abstract

Background: The emergence of ChatGPT, a state-of-the-art language model developed by OpenAI, has introduced a novel avenue for patients to seek medically related information. This technology holds significant promise in terms of accessibility and convenience. However, the use of ChatGPT as a source of accurate information enhancing patient education and engagement requires careful consideration. The objective of this study was to assess the accuracy and reliability of ChatGPT in providing information on the indications and management of complications post-tympanostomy, the most common pediatric procedure in otolaryngology. Methods: We prompted ChatGPT-3.5 with questions and compared its generated responses with the recommendations provided by the latest American Academy of Otolaryngology–Head and Neck Surgery Foundation (AAO-HNSF) “Clinical Practice Guideline: Tympanostomy Tubes in Children (Update)”. Results: A total of 23 responses were generated by ChatGPT against the AAO-HNSF guidelines. Following a thorough review, it was determined that 22/23 (95.7%) responses exhibited a high level of reliability and accuracy, closely aligning with the gold standard. Conclusion: Our research study indicates that ChatGPT may be of assistance to parents in search of information regarding tympanostomy tube insertion and its clinical implications.

## 1. Introduction

Chat GPT is a conversational artificial intelligence (AI) tool designed to be a resource for individuals seeking information on a wide range of topics, including medical issues. One of the tool’s fundamental strengths is its ability to elucidate complex medical terminology and describe complex physiology in a succinct manner [1,2,3,4]. However, concerns pertaining to the accuracy and reliability of the instruction provided by ChatGPT, specifically in matters relating to healthcare, have been raised [5,6,7]. As several parents of children undergoing myringotomy tube insertion in our practice have recently admitted using ChatGPT, the goal of our study was to evaluate the accuracy and efficacy of ChatGPT as a resource for patients seeking information on the most performed otolaryngology surgery: tympanostomy tube insertion. 

We hypothesized that ChatGPT is capable of providing comprehensive answers to questions commonly asked by parents about tympanostomy tube insertion. In order to assess the performance of the application, we evaluated the responses generated by ChatGPT in comparison with the highest standards of healthcare practice. 

The findings of this study can contribute significantly to the ongoing efforts to leverage AI in patient-centered care while also ensuring the provision of high-quality medical information to patients and caregivers. We hope to promote its responsible use as a valuable resource guiding parents to make informed decisions about the care of their child.

It is more important than ever to explore the incorporation of supplementary resources such as AI to enhance the effectiveness of patient–provider communication and the quality of services delivered in the healthcare system.

## 2. Materials and Methods

**Figure 1:**
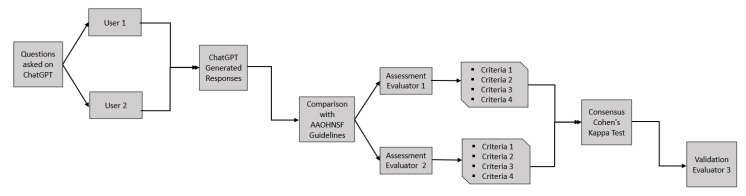
Workflow diagram.

A list of predetermined questions regarding surgical indications and complications post-tympanostomy insertion was presented to ChatGPT by two users on separate devices using the ChatGPT-3.5 version, which is free of charge and readily available to the majority of parents. To prevent any biases from the prior question, a new ChatGPT session was started for each prompt. Responses collected by the two users were recorded in our database to be compared with the statements published in the latest “American Academy of Otolaryngology–Head and Neck Surgery Foundation Clinical Practice Guideline: Tympanostomy Tubes in Children (Update)” [8]. Two independent otolaryngologists evaluated if the answer provided by the chatbot was aligned with the latest guidelines, cited the verbatim guidelines, and explicitly referenced the AAO-HNSF, as well as communicated the importance of seeking a clinician’s input. The inter-rater reliability was assessed using Cohen’s Kappa test. To confirm the consensus on all 4 outputs, the responses of both parties were reviewed by the senior author (SJD). A visual representation of the workflow is depicted in Figure 1. 

## 3. Results

A total of 23 responses generated by ChatGPT were assessed by two otolaryngologists from distinct clinical settings (Table 1). There was perfect agreement between the two judges (Cohen’s K = 100%) that ChatGPT provided comprehensive recommendations that adequately matched the information highlighted by the guidelines.

Both raters agreed that 22/23 responses (95.7%) presented high-quality information closely aligning with the guidelines. Recommendations of one response, pertaining to the need of tympanostomy tube insertion in children with recurrent acute otitis media (AOM) who have unilateral or bilateral middle ear effusion (MEE), differed from the guidelines. The AI tool was not committal as to the treatment provided, stating: “Decision for tympanostomy tube insertion in these cases may depend on factors such as the severity and duration of effusion, impact on hearing, associated symptoms, and overall clinical picture”, whereas the guidelines stipulated that children should be offered bilateral tympanostomy tubes. 

On 2/23 occasions, ChatGPT cited the verbatim AAO-HNSF guideline in its response. Regarding the action point concerning acute tympanostomy tube otorrhea, the question asked on ChatGPT, “Should clinicians prescribe topical antibiotic ear drops for children with uncomplicated acute tympanostomy tube otorrhea?”, cited verbatim the guideline. According to the guidelines from the American Academy of Otolaryngology-Head and Neck Surgery (AAO-HNS), clinicians should consider topical antibiotic ear drops for children with uncomplicated acute tympanostomy tube otorrhea. On a second occasion, regarding the action point concerning water precautions, the question asked on ChatGPT, “Should clinicians encourage routine prophylactic water precautions (earplugs or headbands or avoidance of swimming) for children with tympanostomy tubes?”, cited verbatim the guideline. According to the guidelines from the American Academy of Otolaryngology-Head and Neck Surgery (AAO-HNS), routine prophylactic water precautions, such as the use of earplugs or headbands or avoidance of swimming, are generally not recommended for children with tympanostomy tubes. 

In total, 3/23 of ChatGPT’s responses referenced the AAOHNS guidelines explicitly. ChatGPT’s responses made at least one direct reference to consult with a specialist or healthcare provider when considering all available treatment options and inherent risk factors. These references include the following key examples: “It’s important to have open and thorough discussions with your child’s healthcare provider, usually an ear, nose, and throat specialist (otolaryngologist), to determine the most appropriate course of action for your child”. Another frequently encountered recommendation goes as follows: “It is important for parents to have open communication with the child’s healthcare provider, discuss concerns, and ask questions to ensure a comprehensive understanding of the available options and make informed decisions about their child’s care”.

The evaluators found that the information provided by ChatGPT exceeded the depth of content found in the guidelines and was easily understandable and tailored to meet the needs of parents who may not be familiar with medical terminology.

A comprehensive breakdown of all responses generated by ChatGPT is available as Supplementary Data in Table S1.

## 4. Discussion

ChatGPT is being increasingly used by patients seeking medical opinions [9,10]. Despite the myriad of benefits offered by such technological advancements, it is imperative to ensure the veracity and accuracy of the information provided [9,11]. The fundamental responsibility remains in guaranteeing that the information delivered to patients is trustworthy and aligned with the highest standards of healthcare practice [12,13,14,15].

In this study, the chatbot differed from the guideline in only 1 question out of 23. While the answer provided by ChatGPT was non-committal as to the treatment provided, it remains accurate, and it does not impact safety.

It is important to note that ChatGPT’s answers are subject to modification over time as it acquires new knowledge through learning. The potential risk arises from the reliance on ChatGPT’s capacity to remain updated with the latest guidelines. Implementing follow-up prompts could have provided ChatGPT the opportunity to deliver more direct and accurate responses. A limitation of ChatGPT lies in its ability to capture the tone or context of a prompt and the nuances of a query. Future AI-based clinical decision-making tools must address these limitations.

The current trend toward close integration with search engines and web-based applications is already changing the face of healthcare. It is anticipated that more caregivers will adopt ChatGPT or start using comparable AI models attempting to gain a better understanding of the surgical indications, postoperative complications, and recovery time of various medical treatments and interventions.

In this study, we conducted an evaluation of ChatGPT’s capacity to deliver high-quality responses aimed at augmenting parental education concerning tympanostomy tube insertion in pediatric patients. Our research yields significant insights into its potential advantages for improving patient communication and underscores the importance of its responsible utilization. In light of these findings, it is imperative to consider future implications in medical practice, including the ongoing challenges faced by clinicians and the existing regulatory landscape in healthcare. To further explore the novel application of ChatGPT in otolaryngology practice, additional research should be conducted. 

## 5. Conclusions

Our study suggests that ChatGPT can help parents seeking answers to tympanostomy tube insertion. The comprehensiveness of ChatGPT’s responses is evident in a comparison with the AAOHNSF guideline, where the tool provided predominantly reliable and extended answers, often including ancillary facts and references to support its responses. Although there was a slight discrepancy between the official guideline statement and ChatGPT response in one question, there would be no risk to the child if parents were to follow ChatGPT’s recommendation. In fact, it has helped us develop a pamphlet addressing frequently asked questions by parents. Further research is required to explore the potential of ChatGPT and similar AI tools in improving healthcare delivery and outcomes.

## Figures and Tables

**Table 1:** ChatGPT prompts.

Action Point/Statement (S)	Question Asked on ChatGPT (Q)	Assessment of ChatGPT’s Response (AR)					
			Closely Aligned with Guidelines	Cited AAOHNS *	Explicitly Referenced AAOHNSF *	Recommended Seeking a Clinician	Agreement between Evaluators
S 1: OME of short duration	Q 1: Should physicians perform a tympanostomy tube insertion in children with otitis media effusion (OME) of short duration?	AR 1:	✓			✓	Y
S 2: Hearing evaluation	Q 2: When should clinicians obtain a hearing evaluation for children with otitis media effusion?	AR 2:	✓		✓	✓	Y
S 3: Chronic bilateral OME with hearing difficulty	Q 3: What should clinicians offer for children with chronic bilateral Otitis media effusion (OME) with hearing difficulty for more than 3 months?	AR 3:	✓		✓	✓	Y
S 4: Chronic OME with symptoms	Q 4: What should an otolaryngologist offer to patients with chronic OME with symptoms (balance/vestibular problems, poor school performance, behavioural problems, ear discomfort, reduced quality of life)?	AR 4:	✓			✓	Y
S 5: Surveillance of chronic OME	Q 5: What is the surveillance period required for patients with chronic otitis media effusion who do not receive tympanostomy tubes?	AR 5:	✓		✓	✓	Y
S 6: Recurrent AOM without MEE	Q 6: Should clinicians perform tympanostomy tube insertion for patients with recurrent acute otitis media (AOM) without middle ear effusion (MEE)?	AR 6:	✓			✓	Y
S 7: Recurrent AOM with MEE	Q 7: Should clinicians perform tympanostomy tube insertion for patients with recurrent acute otitis media AOM with unilateral or bilateral middle ear effusion?	AR 7:				✓	Y
S 8: At-risk children	Q 8: Should clinicians determine if a child with recurrent AOM or with OME of any duration is at increased risk for speech, language, or learning problems from otitis media because of baseline sensory, physical, cognitive, or behavioral factors?	AR 8:	✓			✓	Y
S 9: Long-term tubes	Q 9: Should clinicians insert long-term tubes as initial surgery for children who meet criteria for tube insertion?	AR 9:	✓			✓	Y
S 10: Adjuvant adenoidectomy	Q 10: Should clinicians perform adenoidectomy as an adjunct to tympanostomy tube insertion for children to potentially reduce future risk of recurrent otitis media?	AR 10:	✓			✓	Y
S 11: Perioperative education	Q 11: Should clinicians offer education to caregivers of children with tympanostomy in the perioperative section?	AR 11:	✓				
S 12: Perioperative ear drops	Q 12: Should clinicians prescribe peri-operative ear drops after tympanostomy tube placement?	AR 12:	✓			✓	Y
S 13: Acute tympanostomy tube otorrhea	Q 13: Should clinicians prescribe topical antibiotic ear drops for children with uncomplicated acute tympanostomy tube otorrhea?	AR 13:	✓	✓		✓	Y
S 14: Acute tympanostomy tube otorrhea	Q 14: Should clinicians prescribe oral antibiotics for children with uncomplicated acute tympanostomy tube otorrhea?	AR 14:	✓			✓	Y
S 15: Water precautions	Q 15: Should clinicians encourage routine prophylactic water precautions (earplugs or headbands or avoidance of swimming) for children with tympanostomy tubes?	AR 15:	✓	✓		✓	Y
S 16: Follow-up	Q 16: How long should a surgeon wait before examining the ears of a child who had tympanostomy tube insertion?	AR 16:	✓			✓	Y
S 17: Comparison of Short- vs. Long-term Tympanostomy Tubes	Q 17: Comparison of Short vs. Long-Term tympanostomy tubes in terms of duration of ventilation, indications, other common uses, advantages, disadvantages	AR 17:	✓			✓	Y
S 18: Ear tubes recommendation	Q 18: Why are ear tubes recommended?	AR 18:	✓			✓	Y
S 19: Ear tube longevity	Q 19: How long will my child’s ear tubes last?	AR 19:	✓			✓	Y
S 20: Post-op follow-up	Q 20: When does my child need to be seen again after the tubes are placed?	AR 20:	✓			✓	Y
S 21: Complications associated with ear tubes	Q 21: What are the possible complications or problems, or ear tubes?	AR 21:	✓			✓	Y
S 22: Water exposure	Q 22: Does my child need ear plugs when exposed to water?	AR 22:	✓			✓	Y
S 23: Calling ENT	Q 23: When should I call the otolaryngologist (ENT) for my children who had a tympanostomy with ear tubes?	AR 23:	✓			✓	Y

* AAO-HNSF guideline: American Academy of Otolaryngology–Head and Neck Surgery Foundation “Clinical Practice Guideline: Tympanostomy Tubes in Children (Update)”; (✓) is used to indicate, “yes; this criteria has been met”; (Y) indicates “yes, there is a perfect agreement between the two evaluators” on the quality of the ChatGPT’s answer.

## Data Availability

The authors confirm that the data supporting the findings of this study are available within the article and its Supplementary Data file.

## Supplementary Materials

The following supporting information can be downloaded at: https://www.mdpi.com/article/10.3390/children10101634/s1. Table S1: Comprehensive breakdown of ChatGPT generated responses compared to AAO-HNSF guideline.
