# Supplementary material for: Can ChatGPT Guide Parents on Tympanostomy Tube Insertion?
Source: Children (Basel). 2023 Sep 30;10(10):1634. doi: 10.3390/children10101634 (PMC10605420; doi:10.3390/children10101634)
Supplement: Supplementary file 1 [file children-10-01634-s001.zip › children-2598502-supplementary.pdf]

children

Table S1: Comprehensive breakdown of ChatGPT generated responses compared to AAO-HNSF guideline*

Statement	Guideline, Question and Answer
**Statement 1:**<br>**OME of short duration**	**AAO-HNSF Guidelines:**<br>*"Clinicians should not perform tympanostomy tube insertion in children with a single episode of OME of less than 3-month duration, from the date of onset (if known) or from the date of diagnosis (if onset is unknown)."*<br>**Question Asked on ChatGPT:**<br>Should physicians perform a tympanostomy tube insertion in children with otitis media effusion of short duration?<br>**ChatGPT Answer:**<br>The decision to perform a tympanostomy tube insertion (ear tube surgery) in children with otitis media effusion (OME) of short duration can vary depending on several factors. OME refers to the presence of fluid in the middle ear without active infection. Here are some considerations for physicians when determining whether to proceed with ear tube surgery in such cases:<br>• Duration and persistence of OME: While OME of short duration may resolve spontaneously in many cases, if the fluid persists for an extended period (typically three months or more) and is causing significant hearing loss or other related symptoms, ear tube surgery may be considered as a treatment option. Persistent OME can negatively impact a child's hearing, speech development, and overall quality of life.<br>• Severity of symptoms: If a child with short-duration OME experiences significant hearing loss, speech delay, balance problems, or recurrent episodes of acute otitis media (ear infections), ear tube surgery may be recommended to address these issues and improve the child's quality of life.<br>• Impact on daily functioning: If the OME is causing noticeable difficulties in a child's daily activities, such as problems with communication, learning, or behavior, ear tube surgery may be considered to alleviate these challenges and support optimal development.<br>• Individual considerations: Each child is unique, and individual factors such as age, overall health, and the presence of other underlying conditions may influence the decision to proceed with ear tube surgery. The child's healthcare provider will carefully evaluate the specific circumstances and discuss the potential risks and benefits of the procedure.<br>It's important to have open and thorough discussions with your child's healthcare provider, usually an ear, nose, and throat specialist (otolaryngologist), to determine the most appropriate course of action for your child. They will consider the child's specific situation, medical history, and symptoms to guide the decision-making process.<br>In some cases, a trial period of observation or non-surgical interventions may be recommended to assess whether the OME resolves spontaneously or causes significant ongoing issues. Ultimately, the goal is to ensure the best possible outcomes for your child's ear health and overall well-being.<br>**Assessment of ChatGPT's Response:**<br>• Closely aligned with the guidelines<br>• Communicated the importance of seeking a clinician's expertise
**Statement 2:**<br>**Hearing evaluation**	**AAO-HNSF Guidelines:**<br>*"Clinicians should obtain a hearing evaluation if OME persists for 3 months or longer OR prior to surgery when a child becomes a candidate for tympanostomy tube insertion."*<br>**Question Asked on ChatGPT:**

	When should clinicians obtain a hearing evaluation for children with otitis media effusion?<br>**ChatGPT Answer:**<br>Clinicians may consider obtaining a hearing evaluation for children with otitis media effusion (OME) in the following situations:<br>• Persistent or recurrent OME: If a child has persistent or recurrent episodes of OME that last for three months or longer, a hearing evaluation is generally recommended. Prolonged fluid accumulation in the middle ear can cause varying degrees of hearing loss, which may impact a child's speech and language development, academic performance, and overall quality of life.<br>• Concerns about hearing loss or speech delay: If parents or caregivers notice signs of hearing loss or have concerns about their child's speech and language development, a hearing evaluation should be considered. Symptoms of hearing loss in children may include difficulty understanding speech, asking for repetition, not responding to sounds, delayed language development, or changes in behavior.<br>• Risk factors or additional concerns: Children with certain risk factors or additional concerns may benefit from a hearing evaluation, even if the duration of OME is relatively short. Some examples of such risk factors or concerns include a history of recurrent acute otitis media (ear infections), a family history of hearing loss, speech delay, developmental delays, or other medical conditions that can impact hearing.<br>• Impact on academic performance: If a child's OME is affecting their academic performance or if their teacher raises concerns about hearing difficulties in the classroom, it is advisable to pursue a hearing evaluation to assess the extent of the hearing loss and its impact on learning.<br>Hearing evaluations for children are typically performed by audiologists, who specialize in assessing hearing abilities. The evaluation may include various tests, such as pure-tone audiometry, tympanometry, and speech audiometry, to assess the child's hearing sensitivity, middle ear function, and speech understanding abilities.<br>It's important for parents or caregivers to communicate any concerns or observations about their child's hearing to their healthcare provider. The healthcare provider can then determine the need for a hearing evaluation and guide further management based on the child's specific situation.<br>**Assessment of ChatGPT's Response:**<br>• Closely aligned with the guidelines<br>• Explicitly referenced the AAO-HNSF guidelines<br>• Communicated the importance of seeking a clinician's expertise
**Statement 3: Chronic bilateral OME with hearing difficulty**	**AAO-HNSF Guidelines:**<br>*"Clinicians should offer bilateral tympanostomy tube insertion to children with bilateral OME for 3 months or longer AND documented hearing difficulties."*<br>**Question Asked on ChatGPT:**<br>What should clinicians offer for children with chronic bilateral Otitis media effusion with hearing difficulty for more than 3 months?<br>**ChatGPT Answer:**<br>For children with chronic bilateral otitis media effusion (OME) and hearing difficulty persisting for more than three months, clinicians may offer the following interventions:<br>• Tympanostomy tube insertion (ear tube surgery): This is a common intervention for chronic OME with hearing difficulty. The procedure involves the placement of small tubes in the eardrums to help equalize middle ear pressure and improve ventilation, allowing for the drainage of fluid. Ear tube surgery can help restore normal hearing, reduce the frequency of ear infections, and alleviate associated symptoms.

- Hearing aids: If the hearing difficulty is significant and ear tube surgery is not recommended or desired, clinicians may suggest hearing aids. Hearing aids amplify sounds and improve the child's ability to hear and communicate effectively.
- Continued monitoring and reevaluation: In some cases, clinicians may opt for continued monitoring and reevaluation. This approach is suitable for children with mild or moderate OME, where the impact on hearing and quality of life is not severe. Regular check-ups allow clinicians to assess the progress of the condition and determine if any intervention is necessary.

The choice of intervention depends on various factors, including the severity of hearing difficulty, the impact on speech and language development, the persistence of OME, the presence of recurrent infections, and the individual needs and preferences of the child and their family. Clinicians will consider these factors, conduct a comprehensive evaluation, and engage in shared decision-making with the child's parents or guardians to determine the most appropriate intervention.

It is important for parents to have open communication with the child's healthcare provider, discuss concerns, and ask questions to ensure a comprehensive understanding of the available options and make informed decisions about their child's care.

Assessment of ChatGPT's Response:

- Closely aligned with the guidelines
- Explicitly referenced the AAO-HNSF guidelines
- Communicated the importance of seeking a clinician's expertise

---

Statement 4: Chronic OME with symptoms

AAO-HNSF Guidelines:

"Clinicians may perform tympanostomy tube insertion in children with unilateral or bilateral OME for 3 months or longer (chronic OME) AND symptoms that are likely attributable, all or in part, to OME that include, but are not limited to, balance (vestibular) problems, poor school performance, behavioral problems, ear discomfort, or reduced quality of life."

Question Asked on ChatGPT:

What should an otolaryngologist offer to patients with chronic OME with symptoms (balance /vestibular problems, poor school performance, behavioral problems, ear discomfort, reduced quality of life)?

ChatGPT Answer:

For patients with chronic otitis media with effusion (OME) and associated symptoms such as balance/vestibular problems, poor school performance, behavioral problems, ear discomfort, and reduced quality of life, an otolaryngologist may offer the following interventions:

- Tympanostomy tube insertion (ear tube surgery): Ear tube surgery is a common intervention for chronic OME that persists despite conservative management and is causing significant symptoms. The procedure involves the placement of small tubes in the eardrums to improve middle ear ventilation, drainage, and equalization of pressure. Ear tube surgery can help alleviate symptoms, improve hearing, reduce the frequency of ear infections, and enhance overall quality of life.
- Hearing evaluation and management: A comprehensive hearing evaluation, including pure-tone audiometry and other relevant tests, can help assess the impact of OME on hearing. If hearing loss is present, the otolaryngologist may recommend appropriate interventions such as hearing aids or other assistive devices to optimize hearing and address any associated communication difficulties.
- Referral to other specialists: Depending on the specific symptoms and concerns, the otolaryngologist may consider referring the patient to other specialists for further evaluation and management. For example, if balance or vestibular problems are prominent, a referral to a neurologist or vestibular specialist may be warranted to assess and address these issues.

	• Collaborative care: The otolaryngologist may work in collaboration with other healthcare professionals, such as pediatricians, speech therapists, psychologists, and educators, to address the diverse needs of the patient. This interdisciplinary approach can help manage behavioral problems, school performance issues, and provide comprehensive support for the patient's overall well-being.<br>• Symptom management: The otolaryngologist may provide recommendations and prescriptions for symptom management. For example, if the patient experiences ear discomfort, pain relievers or specific ear drops may be prescribed to alleviate the symptoms.<br>It's important for the otolaryngologist to thoroughly assess the patient's symptoms, evaluate their impact on daily functioning and quality of life, and tailor the management approach accordingly. Open communication between the otolaryngologist, patient, and their caregivers is vital for understanding the patient's needs, setting appropriate treatment goals, and ensuring the best possible outcomes.<br>**Assessment of ChaGPT's Response:**<br>• Closely aligned with the guidelines<br>• Communicated the importance of seeking a clinician's expertise
**Statement 5: Surveillance of chronic OME**	**AAO-HNSF Guide-lines:**<br>*"Clinicians should reevaluate, at 3- to 6-month intervals, children with chronic OME who do not receive tympanostomy tubes, until the effusion is no longer present, significant hearing loss is detected, or structural abnormalities of the tympanic membrane or middle ear are suspected."*<br>**Question Asked on ChatGPT:**<br>What is the surveillance period required for patients with chronic otitis media effusion who do not receive tympanostomy tubes?<br>**ChatGPT Answer:**<br>The surveillance period for patients with chronic otitis media effusion (OME) who do not receive tympanostomy tubes can vary based on individual circumstances and the recommendation of the healthcare provider. However, a typical surveillance period for these patients may involve regular follow-up appointments at intervals determined by the healthcare provider. The purpose of surveillance is to monitor the patient's condition, assess any changes, and determine if further intervention is necessary. Here are some general considerations:<br>• Initial follow-up: After the diagnosis of chronic OME, the healthcare provider will typically schedule an initial follow-up appointment to evaluate the patient's symptoms, hearing status, and the impact of OME on their daily life. This initial appointment may occur within a few weeks to a couple of months from the diagnosis.<br>• Subsequent follow-up appointments: The frequency of subsequent follow-up appointments can vary based on the patient's age, symptoms, and the severity of OME. In some cases, follow-up visits may be scheduled every few months or every 3-6 months to monitor the progression of the condition and assess any changes in symptoms or hearing.<br>• Ongoing monitoring: The healthcare provider will continue to assess the patient's symptoms, hearing, and quality of life during each follow-up appointment. They may conduct a physical examination of the ears and evaluate the patient's response to previous management strategies. The duration of the surveillance period will depend on the individual patient's progress, with adjustments made as necessary.<br>• Trigger for intervention: During the surveillance period, the healthcare provider will closely monitor any changes in the patient's condition. If the OME persists or worsens, if symptoms significantly impact the patient's quality of life, or if there are concerns about hearing loss or developmental delays, the healthcare provider may recommend further interventions, such as tympanostomy tube insertion or alternative treatments.

	It's important for patients and their caregivers to communicate openly with the healthcare provider, report any changes in symptoms, and attend regular follow-up appointments as scheduled. The healthcare provider will determine the appropriate surveillance period and follow-up schedule based on the patient's unique circumstances and response to treatment.<br>**Assessment of ChatPPT's Response:**<br>• Closely aligned with the guidelines<br>• Explicitly referenced the AAO-HNSF guidelines<br>• Communicated the importance of seeking a clinician's expertise
**Statement 6: Recurrent AOM without MEE**	**AAO-HNSF Guide-lines:**<br>*"Clinicians should not perform tympanostomy tube insertion in children with recurrent AOM who do not have MEE in either ear at the time of assessment for tube candidacy."*<br>**Question Asked on ChatGPT:**<br>Should clinicians perform tympanostomy tube insertion for patients with recurrent AOM without middle ear effusion?<br>**ChatGPT Answer:**<br>The decision to perform tympanostomy tube insertion for patients with recurrent acute otitis media (AOM) without middle ear effusion is a topic of debate among healthcare professionals, and there is no consensus in clinical guidelines regarding this specific scenario. The need for tympanostomy tubes primarily arises when there is persistent middle ear effusion, as tubes help ventilate the middle ear and promote the clearance of fluid.<br>In cases of recurrent AOM without middle ear effusion, the management approach may focus on alternative strategies to prevent or reduce the frequency of future AOM episodes. These strategies may include:<br>• Addressing underlying risk factors: Identifying and addressing any underlying risk factors associated with recurrent AOM, such as exposure to secondhand smoke, attending daycare, or anatomical abnormalities, can help reduce the frequency of episodes.<br>• Antibiotic prophylaxis: In some cases, healthcare providers may consider prescribing antibiotic prophylaxis, which involves giving the patient low-dose antibiotics over an extended period to prevent future AOM episodes. However, this approach is not without controversy and should be carefully considered on an individual basis.<br>• Immunizations: Ensuring the patient is up to date on immunizations, including the pneumococcal conjugate vaccine and influenza vaccine, can help reduce the risk of AOM.<br>• Patient education and close monitoring: Providing education to patients and their caregivers about preventive measures, such as proper hand hygiene and avoidance of known triggers, can be beneficial. Close monitoring of the patient's condition through regular follow-up visits allows for early detection and intervention if the frequency or severity of AOM episodes changes.<br>Ultimately, the decision regarding tympanostomy tube insertion for patients with recurrent AOM without middle ear effusion should be made on an individual basis, considering the patient's clinical presentation, history, associated risk factors, and consultation with an otolaryngologist or ear, nose, and throat specialist. The healthcare provider will weigh the potential benefits and risks of tube insertion and consider alternative management strategies in order to provide the most appropriate care for the patient.<br>**Assessment of ChatGPT's Response:**<br>• Closely aligned with the guidelines<br>• Communicated the importance of seeking a clinician's expertise
**Statement 7: Recurrent AOM with MEE**	***AAO-HNSF Guidelines:***<br>*"Clinicians should offer bilateral tympanostomy tube insertion in children with recurrent AOM who have unilateral or bilateral MEE at the time of assessment for tube candidacy."*<br>**Question Asked on ChatGPT:**

Should clinicians perform tympanostomy tube insertion for patients with recurrent AOM with unilateral or bilateral middle ear effusion?

ChatGPT Answer:

The decision to perform tympanostomy tube insertion for patients with recurrent acute otitis media (AOM) and unilateral or bilateral middle ear effusion should be based on careful evaluation of the individual patient's clinical presentation and consultation with an otolaryngologist or ear, nose, and throat specialist. Here are some considerations:

- Bilateral middle ear effusion: Bilateral middle ear effusion refers to fluid accumulation in both ears. It may be more likely to cause hearing loss and impact speech and language development. Tympanostomy tube insertion is often considered if bilateral effusion persists despite appropriate medical management and impacts the patient's hearing, quality of life, or development.
- Unilateral middle ear effusion: Unilateral middle ear effusion, where fluid accumulates in only one ear, may still cause hearing loss and affect the patient's well-being. The decision for tympanostomy tube insertion in these cases may depend on factors such as the severity and duration of effusion, impact on hearing, associated symptoms, and overall clinical picture.
- Frequency and severity of AOM episodes: The recurrence and severity of AOM episodes play a role in the decision-making process. If the patient experiences frequent AOM episodes with associated middle ear effusion, tympanostomy tube insertion may be considered to reduce the risk of future infections and their impact on the ear.
- Impact on hearing and development: Persistent or recurrent middle ear effusion can lead to hearing loss, especially in young children during critical periods of language development. If the effusion is causing significant hearing impairment or developmental delays, tympanostomy tube insertion may be recommended to restore hearing and support normal development.
- Individual patient factors: Various individual patient factors should be taken into account, including age, overall health, previous treatment response, and parental preferences. Shared decision-making between the healthcare provider, patient, and their family is essential in determining the most appropriate course of action.

The decision to perform tympanostomy tube insertion is not universal for all patients with recurrent AOM and middle ear effusion. It requires careful assessment, consideration of the patient's specific circumstances, and professional judgment. Consulting with an otolaryngologist or ear, nose, and throat specialist will help guide the decision-making process and ensure the best possible care for the patient.

Assessment of ChatGPT's Response:

- Communicated the importance of seeking a clinician expertise

---

Statement 8:
At-risk children

AAO-HNSF Guidelines:

"Clinicians should determine if a child with recurrent AOM or with OME of any duration is at increased risk for speech, language, or learning problems from otitis media because of baseline sensory, physical, cognitive, or behavioral factors."

Question Asked on ChatGPT:

Should clinicians determine if a child with recurrent AOM or with OME of any duration is at increased risk for speech, language, or learning problems from otitis media because of baseline sensory, physical, cognitive, or behavioral factors?

ChatGPT Answer:

Yes, clinicians should consider various factors to determine if a child with recurrent acute otitis media (AOM) or chronic otitis media with effusion (OME) is at increased risk for speech, language, or learning problems. These factors include baseline sensory, physical,

cognitive, or behavioral factors that may influence the child's development and educational outcomes. Evaluating these factors can help identify children who may require additional support or intervention. Here are some key considerations:

- Sensory factors: Children with pre-existing sensory impairments, such as hearing loss or visual impairments, may be at higher risk for speech, language, or learning problems due to the added impact of otitis media. Clinicians should assess the child's sensory status and consider the cumulative effect of sensory impairments on their development.
- Physical factors: Certain physical conditions, such as structural abnormalities of the ear or craniofacial anomalies, can increase the risk of otitis media and its impact on speech, language, or learning. Clinicians should evaluate the child's physical health and any anatomical factors that may affect their ear health or communication abilities.
- Cognitive factors: Children with underlying cognitive impairments, developmental delays, or neurodevelopmental disorders may be more susceptible to the adverse effects of otitis media on speech, language, or learning. Clinicians should assess the child's cognitive abilities and consider how otitis media may interact with their existing cognitive profile.
- Behavioral factors: Behavioral factors, such as attention difficulties, hyperactivity, or disruptive behaviors, can impact a child's ability to cope with the challenges posed by otitis media and its potential impact on speech, language, or learning. Clinicians should consider the child's behavioral functioning and its potential impact on their overall development.

By evaluating these baseline factors, clinicians can better understand the individual risk profile of a child with recurrent AOM or OME and make informed decisions about appropriate interventions or support services. It is important for clinicians to conduct a comprehensive assessment, collaborate with other professionals as needed, and provide appropriate referrals for further evaluation or intervention when necessary.

Assessment of ChatGPT's Response:

- Closely aligned with guidelines
- Communicated the importance of seeking a clinician's expertise

---

Statement 9: Long-term tubes

AAO-HNSF Guidelines:

"The clinician should not place long-term tubes as initial surgery for children who meet criteria for tube insertion unless there is a specific reason based on an anticipated need for prolonged middle ear ventilation beyond that of a short-term tube."

Question Asked on ChatGPT:

Should clinicians insert long term tubes as initial surgery for children who meet criteria for tube insertion?

ChatGPT Answer:

The decision to insert long-term tubes as the initial surgery for children who meet the criteria for tube insertion depends on several factors, including the child's specific condition, the severity of symptoms, and the healthcare provider's clinical judgment. While short-term tubes are often considered as the initial intervention, long-term tubes may be appropriate in certain cases. Here are some considerations:

- Severity and frequency of otitis media: If a child has a history of severe or recurrent acute otitis media (AOM) episodes that significantly impact their quality of life, long-term tubes may be considered as the initial intervention. Long-term tubes provide continuous ventilation and drainage of the middle ear, reducing the likelihood of future AOM episodes.
- Chronic otitis media with effusion (OME): If the child has chronic OME with persistent middle ear fluid that does not resolve despite conservative management, long-

	term tubes may be considered to alleviate symptoms, improve hearing, and prevent complications.<br>• Impact on speech, language, or learning: If the child's speech, language, or learning development is at risk due to the recurrent or chronic nature of their otitis media, long-term tubes may be considered to ensure optimal hearing and minimize the impact on their educational progress.<br>• Individual patient factors: The decision should also take into account individual patient factors such as age, overall health, previous treatment response, and parental preferences. Shared decision-making between the healthcare provider, patient, and their family is important in determining the most appropriate course of action.<br>It is essential for clinicians to thoroughly evaluate each child's unique circumstances and consider the risks and benefits of different treatment options. Collaboration with an otolaryngologist or ear, nose, and throat specialist can provide further guidance in determining the most suitable surgical approach for children who meet the criteria for tube insertion.<br>**Assessment of ChatGPT's Response:**<br>• Closely aligned with the guidelines<br>• Communicated the importance of seeking a clinician's expertise
**Statement 10: Adjuvant adenoidectomy**	**AAO-HNSF Guidelines:**<br>*"Clinicians may perform adenoidectomy as an adjunct to tympanostomy tube insertion for children with symptoms directly related to the adenoids (adenoid infection or nasal obstruction) OR in children aged 4 years or older to potentially reduce future incidence of recurrent otitis media or the need for repeat tube insertion."*<br>**Question Asked on ChatGPT:**<br>Should clinicians perform adenoidectomy as an adjunct to tympanostomy tube insertion for children to potentially reduce future risk of recurrent otitis media?<br>ChatGPT Answer:<br>The decision to perform an adenoidectomy as an adjunct to tympanostomy tube insertion for children to potentially reduce the future risk of recurrent otitis media should be made based on careful consideration of the individual patient's condition and in consultation with an otolaryngologist or ear, nose, and throat specialist. The use of adenoidectomy in combination with tube insertion is a topic of debate and the guidelines may vary.<br>Traditionally, adenoidectomy has been considered as a treatment option for children with recurrent acute otitis media (AOM) or chronic otitis media with effusion (OME) who also have enlarged adenoids. The rationale behind adenoidectomy is that the adenoids, which are lymphoid tissues located at the back of the nose, can contribute to the obstruction of the Eustachian tubes and impair proper drainage of the middle ear.<br>Some factors to consider regarding the potential benefit of adenoidectomy as an adjunct to tube insertion include:<br>• Enlarged adenoids: Adenoidectomy may be more beneficial in children who have significant enlargement of the adenoids, which can be observed through physical examination or imaging studies.<br>• Recurrent AOM or chronic OME: Adenoidectomy as an adjunct to tube insertion may be considered in children with a history of frequent or severe AOM episodes or chronic OME that persists despite conservative management.<br>• Other factors: The decision may also consider other factors such as the child's age, overall health, response to previous treatments, and individual risk factors.<br>It's important to note that the use of adenoidectomy as an adjunct to tube insertion is not universally recommended in all cases. The decision should be made on a case-by-case basis, taking into account the specific circumstances and in consultation with a qualified

healthcare professional who can evaluate the child's condition and provide personalized recommendations based on the best available evidence and clinical expertise.

Assessment of ChatGPT's Response:

- Closely aligned with the guidelines
- Communicated the importance of seeking a clinician's expertise

---

Statement 11: Perioperative education

AAO-HNSF Guide-lines:

"In the perioperative period, clinicians should educate caregivers of children with tympanostomy tubes."

Question Asked on ChatGPT:

Should clinicians offer education to caregivers of children with tympanostomy in the peri-operative section?

ChatGPT Answer:

Yes, clinicians should offer education to caregivers of children undergoing tympanostomy tube insertion in the peri-operative period. Providing education and information to caregivers is an important aspect of the overall care for the child. Here are some key points that clinicians may cover during the peri-operative education:

- Procedure explanation: Clinicians should explain the tympanostomy tube insertion procedure in a clear and understandable manner. They can describe the purpose of the surgery, how the tubes will be placed, and the expected benefits for the child.
- Risks and complications: Caregivers should be informed about the potential risks and complications associated with the procedure. This may include possible bleeding, infection, tube blockage, or tube extrusion. It's important to address any concerns or questions the caregivers may have.
- Anesthesia and recovery: If the procedure requires anesthesia, caregivers should be in-formed about the type of anesthesia that will be used and what to expect during the recovery period. They should be educated about post-operative care, pain management, and any restrictions or precautions that need to be followed.
- Follow-up appointments: Caregivers should be informed about the need for follow-up appointments after the surgery. This may include scheduling visits to check the placement and function of the tubes and to monitor the child's ear health.
- Care instructions: Caregivers should receive instructions on how to care for the child's ears and the tubes post-surgery. This may include guidance on keeping the ears dry during bathing or swimming, using earplugs if necessary, and how to handle any discharge or concerns related to the tubes.
- Expected outcomes: Caregivers should have realistic expectations regarding the benefits of tympanostomy tubes, such as improved hearing, reduced risk of ear infections, and improved quality of life for the child. It's important to discuss any limitations or potential long-term considerations.
- Addressing concerns: Caregivers should be encouraged to ask questions and express any concerns they may have. Clinicians should provide clear channels of communication for ongoing support and address any additional information or clarification needed.

By offering comprehensive education and addressing caregivers' questions and concerns, clinicians can help ensure that caregivers are well-informed and prepared to support the child's care during the peri-operative period and beyond.

Assessment of ChatGPT's Response:

- Closely aligned with the guidelines

Statement 12: Perioperative ear drops

AAO-HNSF Guide-lines:

"Clinicians should not routinely prescribe postoperative antibiotic ear drops after tympanostomy tube placement."

Question Asked on ChatGPT:

Should clinicians prescribe perio-operative ear drops after tympanostomy tube placement?

ChatGPT Answer:

The decision to prescribe peri-operative ear drops after tympanostomy tube placement may vary depending on the specific circumstances and clinical judgment of the healthcare provider. In general, the use of ear drops after tube placement is not a routine recommendation, but there are situations where they may be beneficial. Here are some factors to consider:

- Ear discharge or infection: If the child has active ear discharge or signs of infection before or after tube placement, the clinician may prescribe antibiotic ear drops to help treat the infection and promote healing.
- Otitis externa prevention: In some cases, especially if the child is prone to developing outer ear infections (otitis externa), clinicians may prescribe antibiotic or antiseptic ear drops as a preventive measure to reduce the risk of infection in the external ear canal.
- Otitis media with effusion (OME): If the primary reason for tube placement is chronic OME, without signs of infection, the routine use of ear drops may not be necessary. Instead, regular follow-up visits to monitor the middle ear and tubes may be recommended.
- Caregiver preference: Caregivers' preferences and expectations can also influence the decision. Some caregivers may feel more comfortable using ear drops as part of the post-operative care, even if they are not medically necessary.

It's important for caregivers to follow the specific instructions provided by the healthcare provider regarding post-operative care. This may include keeping the ears dry during bathing or swimming and avoiding the use of earplugs or other objects that can block the tubes.

Ultimately, the decision to prescribe peri-operative ear drops should be based on the individual needs of the child, the presence of any ear infection or discharge, and the clinical judgment of the healthcare provider. Caregivers should consult with the healthcare provider to determine the appropriate post-operative care plan for their child.

Assessment of ChatGPT's Response:

- Closely aligned with the guidelines
- Communicated the importance of seeking a clinician expertise

---

Statement 13: Acute tympanostomy tube otorrhea

AAO-HNSF Guide-lines:

"Clinicians should prescribe topical antibiotic ear drops only, without oral antibiotics, for children with uncomplicated acute tympanostomy tube otorrhea."

Question Asked on ChatGPT:

Should clinicians prescribe topical antibiotic ear drops for children with uncomplicated acute tympanostomy tube otorrhea?

ChatGPT Answer:

According to the guidelines from the American Academy of Otolaryngology-Head and Neck Surgery (AAO-HNS), clinicians should consider topical antibiotic ear drops for children with uncomplicated acute tympanostomy tube otorrhea. Uncomplicated acute tympanostomy tube otorrhea refers to cases where there is no severe infection or systemic symptoms present. The decision to prescribe topical antibiotic ear drops should be made based on the severity of symptoms and the individual patient's clinical presentation.

Here are some key considerations:

- Clinical assessment: The healthcare provider should evaluate the child's symptoms and conduct a thorough examination of the ear to determine the severity and nature of the otorrhea (ear drainage). This assessment helps to rule out more severe infections or complications that may require systemic antibiotics or further interventions.

	• Mild to moderate otorrhea: Topical antibiotic ear drops are typically recommended for children with mild to moderate otorrhea, where the discharge is not excessive and there are no signs of severe infection.<br>• Antibiotic selection: The choice of antibiotic ear drops may depend on the suspected or confirmed bacterial pathogens in the otorrhea. Commonly used antibiotic drops include fluoroquinolones (e.g., ciprofloxacin) or aminoglycosides (e.g., neomycin).<br>• Duration of treatment: The duration of treatment with topical antibiotic ear drops may vary but is typically recommended for a limited period, such as 7 to 10 days. This helps to control the infection and promote healing.<br>It's important to note that the decision to prescribe topical antibiotic ear drops should be made by a qualified healthcare provider based on a comprehensive assessment of the child's condition. Caregivers should follow the healthcare provider's instructions regarding the use of ear drops, including proper administration techniques and the duration of treatment.<br>It is advisable to consult with a healthcare professional to get specific and personalized recommendations based on the child's individual situation and the most up-to-date guidelines.<br>**Assessment of ChatGPT's Response:**<br>• Closely aligned with the guidelines<br>• Cited the verbatim AAO-HNS guidelines<br>• Communicated the importance of seeking a clinician expertise
**Statement 14: Acute tympanostomy tube otorrhea**	**AAO-HNSF Guide-lines:**<br>*"Clinicians should prescribe topical antibiotic ear drops only, without oral antibiotics, for children with uncomplicated acute tympanostomy tube otorrhea."*<br>**Question Asked on ChatGPT:**<br>Should clinicians prescribe oral antibiotics for children with uncomplicated acute tympanostomy tube otorrhea?<br>**ChatGPT Answer:**<br>The decision to prescribe oral antibiotics for children with uncomplicated acute tympanostomy tube otorrhea (ear drainage) depends on several factors and should be made by a healthcare provider based on their assessment of the individual case. Here are some key considerations:<br>• Evaluation of the otorrhea: The healthcare provider will assess the characteristics of the ear drainage, including its color, consistency, and odor. They will also examine the ear to determine if there are signs of infection or other complications. If the otorrhea is mild and the child is not experiencing significant symptoms or complications, oral antibiotics may not be necessary.<br>• Severity and duration of symptoms: If the otorrhea is accompanied by signs of infection, such as pain, redness, swelling, or fever, or if the symptoms are persistent or worsening, the healthcare provider may consider prescribing oral antibiotics to help treat the infection and prevent further complications.<br>• Risk of complications: Certain factors may increase the risk of complications from otorrhea, such as the presence of a known bacterial infection, a history of recurrent infections, or underlying medical conditions that weaken the immune system. In such cases, the healthcare provider may be more likely to prescribe oral antibiotics to minimize the risk of complications. Individual patient factors:<br>The healthcare provider will consider the child's age, medical history, previous response to treatments, and any specific concerns or risk factors when deciding whether to prescribe oral antibiotics. It's important to note that not all cases of uncomplicated acute tympanostomy tube otorrhea require oral antibiotic treatment. In some cases, the otorrhea

may resolve on its own without intervention. However, if the healthcare provider determines that oral antibiotics are necessary, they will prescribe the appropriate medication and provide instructions on its use.

If you have concerns about your child's otorrhea or need specific advice regarding treatment, it is recommended to consult with the healthcare provider who is familiar with your child's case. They can assess the situation, provide personalized recommendations, and determine the most appropriate course of action.

Assessment of ChatGPT's Response:

- Closely aligned with the guidelines
- Communicated the importance of seeking a clinician expertise

---

Statement 15: Water precautions

AAO-HNSF Guide-lines:

"Clinicians should not encourage routine, prophylactic water precautions (use of earplugs or headbands, avoidance of swimming or water sports) for children with tympanostomy tubes."

Question Asked on ChatGPT:

Should clinicians encourage routine prophylactic water precautions (earplugs or headbands or avoidance of swimming) for children with tympanostomy tubes?

ChatGPT Answer:

According to the guidelines from the American Academy of Otolaryngology-Head and Neck Surgery (AAO-HNS), routine prophylactic water precautions, such as the use of earplugs or headbands or avoidance of swimming, are generally not recommended for children with tympanostomy tubes. However, the decision regarding water precautions should be made on an individual basis, taking into account the specific circumstances and recommendations from the healthcare provider. Here are some key points to consider:

- Consultation with the healthcare provider: It is important for caregivers to consult with the healthcare provider who performed the tube insertion procedure or an otolaryngologist for personalized guidance regarding water precautions. They can evaluate the child's specific situation and provide tailored recommendations based on the child's medical history, the type of tubes inserted, and any associated risks.
- Balanced approach: The AAO-HNS guidelines emphasize the importance of maintaining a balanced approach to water exposure. While routine use of earplugs or headbands or complete avoidance of swimming may not be necessary for most children, certain precautions may be advised in specific situations. For example, if the child is prone to frequent outer ear infections (otitis externa) or has a history of complications related to water exposure, specific recommendations may be given to minimize the risk.
- Individual risk factors: Consideration should be given to individual risk factors, such as the presence of chronic otorrhea (persistent ear drainage), recurrent infections, or other underlying medical conditions that may increase the risk of complications from water exposure. In such cases, additional precautions may be recommended.
- Informed decision-making: Caregivers should be educated about the potential risks and benefits of water exposure and the specific factors to consider in their child's case. They should be empowered to make informed decisions in collaboration with the healthcare provider, taking into account the child's overall health, lifestyle, and preferences.

It is important to note that even with water precautions, complete prevention of water entry into the ears is not always guaranteed. Caregivers should monitor for any signs of infection or discomfort and promptly seek medical attention if any concerns arise.

Ultimately, the decision regarding water precautions should be made on an individual basis, considering the specific needs and risks of the child. Consulting with the healthcare provider is crucial to determine the most appropriate approach for the child with tympanostomy tubes.

	**Assessment of ChatGPT's Response:**<br>• Closely aligned with the guidelines<br>• Cited the verbatim AAO-HNS guidelines<br>• Communicated the importance of seeking a clinician's expertise
**Statement 16: Follow-up**	**AAO-HNSF Guide-lines:**<br>*"The surgeon or designee should examine the ears of a child within 3 months of tympanostomy tube insertion AND should educate families regarding the need for routine, periodic follow-up to examine the ears until the tubes extrude."*<br>**Question Asked on ChatGPT:**<br>How long should a surgeon wait before examining the ears of a child who had tympanostomy tube insertion?<br>**ChatGPT Answer:**<br>The timing for a post-operative examination of the ears after tympanostomy tube insertion can vary based on the specific circumstances and the preferences of the surgeon. However, in general, a post-operative examination is typically conducted within the first 1 to 2 weeks after the surgery. This initial follow-up visit allows the surgeon to assess the healing of the surgical site, evaluate the placement and function of the tubes, and address any concerns or questions from the caregivers.<br>During this examination, the surgeon will use an otoscope or an operating microscope to visualize the ears and the tubes. They will check the positioning of the tubes, ensure that they are patent (open), and assess the overall condition of the ears.<br>It's important for caregivers to attend this initial post-operative visit as scheduled and to follow any specific instructions provided by the surgeon. Depending on the child's progress and the surgeon's assessment, additional follow-up visits may be scheduled at regular intervals to monitor the ears, the function of the tubes, and the overall ear health.<br>It's worth noting that the exact timing and frequency of follow-up examinations may vary based on individual factors such as the specific surgical technique, the child's condition, and any unique considerations identified by the surgeon. It's best to consult with the surgeon or healthcare provider who performed the tympanostomy tube insertion for specific instructions regarding the timing of post-operative examinations for your child.<br>**Assessment of ChatGPT's Response:**<br>• Closely aligned with the guidelines<br>• Communicated the importance of seeking a clinician's expertise
**Statement 17: Comparison of Short- vs Long-term Tympanostomy Tubes**	**AAO-HNSF Guide-lines*:***<br>• *Duration of Ventilation:*<br>o *Short-term tubes:8 to 18 months, on average; some stay in place 3 years or longer*<br>o *Long-term tubes: 15 months or longer, usually 2 to 3 years; some stay in place 5 years or longer*<br>• *Indications:*<br>o *Short-term tubes: Routine choice for first ear tube surgery for a child*<br>o *Long-term tubes: Selective choice for first ear tube surgery for a child when a need for prolonged ventilation is anticipated; option for repeat tube surgery*<br>• *Other Common Uses:*<br>o *Short-term tubes: Repeat ear tube surgery in children without significant risk factors or anatomic changes in the eardrum that would warrant a long-term tube.*<br>o *Long-term tubes: Cleft palate, stenotic ear canal, abnormal tympanic membrane (atrophy, atelectasis, retraction pocket), premature extrusion of multiple prior short-term tubes.*<br>• *Advantages:*<br>o *Short-term tubes: Reduce incidence (vs long-term tubes) of perforation, chronic otorrhea, granulation tissues, myringosclerosis, ease of insertion*

  - *Long-term tubes: Longer period of middle ear ventilation, potential to avoid repeat insertion of short-term tubes and exposure to general anesthesia; ability to insert silastic T-tub in stenotic canal*
- *Disadvantages:*
  - *Short-term tubes: Potential for early extrusion and need for repeat tube surgery*
  - *Long-term tubes: Increased incidence (vs short-term tube) of perforation, chronic otorrhea, granulation tissue, myringosclerosis, medialization (if short shaft with no outer flange); more difficult to insert."*

Question Asked on ChatGPT:

Comparison of Short vs. Long-Term tympanostomy tubes in terms of duration of ventilation, indications, other common uses, advantages, disadvantages

ChatGPT Answer:

Here's a comparison between short-term and long-term tympanostomy tubes:

- Duration of Ventilation:
  - Short-term tubes: These tubes have a shorter duration of ventilation and typically stay in place for about 6 to 12 months before they naturally extrude or fall out on their own.
  - Long-term tubes: Also known as ventilation tubes or grommets, long-term tubes are designed to stay in place for a longer period, usually more than 12 months. Some types of long-term tubes can remain in the ears for several years.
- Indications:
  - Short-term tubes: Short-term tubes are commonly used for conditions such as recurrent acute otitis media (AOM), otitis media with effusion (OME), or persistent middle ear fluid that has not resolved with other treatments.
  - Long-term tubes: Long-term tubes are typically recommended for children with chronic or persistent middle ear problems, such as chronic OME or recurrent AOM, that have not responded to other conservative treatments. They may also be considered for children with specific risk factors or underlying conditions that increase the likelihood of ongoing middle ear issues.
- Other Common Uses:
  - Short-term tubes: Besides treating middle ear infections and fluid, short-term tubes can be used for diagnostic purposes during certain procedures or surgeries, allowing for direct visualization of the middle ear.
  - Long-term tubes: In addition to treating chronic middle ear problems, long-term tubes can be used to manage conditions like Eustachian tube dysfunction, conductive hearing loss, and to facilitate delivery of topical medications to the middle ear.
- Advantages:
  - Short-term tubes: Short-term tubes provide temporary relief and ventilation of the middle ear, helping to equalize pressure, drain fluid, and reduce the frequency of ear infections.
  - Long-term tubes: Long-term tubes offer more sustained ventilation and drainage, helping to prevent the accumulation of fluid, improve hearing, and reduce the frequency of ear infections over an extended period.
- Disadvantages:
  - Short-term tubes: The main disadvantage of short-term tubes is that they have a finite lifespan and eventually need to be replaced or naturally extruded, requiring another surgical procedure for tube insertion if ongoing middle ear problems persist.
  - Long-term tubes: The main drawback of long-term tubes is that they may remain in place longer than necessary, potentially leading to persistent perforation of the

	eardrum, tube blockage, or other complications. Additionally, long-term tubes may require monitoring and follow-up visits over an extended period.<br>It's important to note that the choice between short-term and long-term tubes depends on the individual's specific condition, severity of symptoms, and the healthcare provider's judgment. The decision should be made after careful consideration of the child's medical history, risk factors, and the potential benefits and risks associated with each option. Consulting with an otolaryngologist or ENT specialist is crucial to determine the most appropriate type of tube for an individual case.<br>**Assessment of ChatGPT's Response:**<br>• Closely aligned with the guidelines<br>• Communicated the importance of seeking a clinician's expertise
**Statement 18: Ear tubes recommendation**	**AAO-HNSF Guide-lines:**<br>*"Ear tubes are recommended for frequent ear infections or prolonged fluid in the ears. They will:*<br>• *Help decrease the number of ear infections*<br>• *Allow any future ear infections to be treated with antibiotic ear drops instead of antibiotics that are taken by mouth*<br>• *Help prevent fluid from backing up into the area behind the eardrum (middle ear)*<br>• *Improve hearing that is decreased because of fluid in the middle ear."*<br>**Question Asked on ChatGPT:**<br>Why are ear tubes recommended?<br>**ChatGPT Answer:**<br>Ear tubes, also known as tympanostomy tubes or grommets, are recommended for various reasons related to middle ear problems. Here are some common reasons why ear tubes may be recommended:<br>• Recurrent Acute Otitis Media (AOM): Ear tubes can be recommended for children who experience frequent episodes of acute otitis media (middle ear infection). By placing tubes, the drainage of fluid from the middle ear is improved, reducing the likelihood of fluid buildup and recurrent infections.<br>• Otitis Media with Effusion (OME): OME refers to the presence of persistent fluid in the middle ear without active infection. If OME persists despite other treatment options, such as antibiotics or nasal decongestants, ear tubes may be recommended to help ventilate the middle ear and facilitate fluid drainage.<br>• Chronic Otitis Media: In cases where the middle ear infection becomes chronic, with ongoing fluid and inflammation, ear tubes can be used to manage the condition. By providing ventilation and drainage, ear tubes help reduce the severity and frequency of infections.<br>• Conductive Hearing Loss: Fluid accumulation in the middle ear can cause temporary hearing loss. Ear tubes help equalize pressure, drain fluid, and improve hearing by restoring normal middle ear function.<br>• Eustachian Tube Dysfunction: The Eustachian tubes are responsible for equalizing pressure between the middle ear and the throat. Dysfunction of these tubes can lead to chronic fluid buildup and recurrent infections. Ear tubes can help bypass Eustachian tube dysfunction and maintain proper middle ear ventilation.<br>• Certain Risk Factors: Children with specific risk factors, such as cleft palate, Down syndrome, or other craniofacial abnormalities, may be more prone to middle ear problems. In such cases, ear tubes may be recommended as a preventive measure or to manage ongoing issues.<br>The primary goal of ear tube placement is to improve ventilation, drainage, and prevent complications associated with middle ear problems. By promoting proper middle ear

	function, ear tubes can help reduce the frequency of infections, improve hearing, and alleviate symptoms like ear pain or pressure.<br>**Assessment of ChatGPT's Response:**<br>• Closely aligned with the guidelines<br>• Communicated the importance of seeking a clinician's expertise
**Statement 19:**<br>**Ear tube longevity**	**AAO-HNSF Guide-lines:**<br>*"Most ear tubes last about 6 to 18 months. By the time the tube comes out about 80% of children will have much better ear function and will not need to have the tube replaced."*<br>**Question Asked on ChatGPT:**<br>How long will my child's ear tubes last?<br>**ChatGPT Answer:**<br>The duration that ear tubes (tympanostomy tubes) remain in place can vary for each child. Generally, there are two types of ear tubes: short-term tubes and long-term tubes.<br>• Short-term tubes: These tubes are designed to stay in place for a shorter duration, typically around 6 to 12 months. After this period, they naturally extrude or fall out on their own. Short-term tubes are commonly used for conditions like recurrent acute otitis media (AOM) or otitis media with effusion (OME) that have not resolved with other treatments.<br>• Long-term tubes: Also known as ventilation tubes or grommets, long-term tubes are intended to remain in the ears for a longer period, often more than 12 months. Some types of long-term tubes can stay in place for several years. They are typically recommended for children with chronic or persistent middle ear problems, such as chronic OME or recurrent AOM, that have not responded to other conservative treatments.<br>It's important to note that the duration of tube placement can vary among individuals. Factors such as the child's specific condition, their ear anatomy, the type of tube used, and their overall health can influence how long the tubes remain in place. Some children may experience early extrusion, while others may require tube removal or replacement if ongoing middle ear issues persist.<br>Regular follow-up visits with the healthcare provider who inserted the tubes are essential to monitor the tube's function, assess the middle ear status, and determine the appropriate timing for tube removal or replacement. The healthcare provider will evaluate the child's progress and decide if and when the tubes need to be removed based on the individual's specific needs.<br>It's important for caregivers to attend scheduled follow-up visits, communicate any concerns or changes in symptoms, and follow the healthcare provider's recommendations regarding the duration of tube placement and any necessary interventions.<br>**Assessment of ChatGPT's Response:**<br>• Closely aligned with the guidelines<br>• Communicated the importance of seeking a clinician's expertise
**Statement 20:**<br>**Post-op follow-up**	**AAO-HNSF Guidelines:**<br>*"After Surgery: We will see your child within 3 months to make sure that the ear tubes are in place and working. We often check your child's hearing at that visit.*<br>*Ongoing Follow-Up: After this first visit, we should see your child regularly, usually every 6 months, while the tubes are in the ears to make sure that the tubes are working and to check for any possible problems, as discussed in the next section. Keep in mind that regular follow-up visits are important, even if your child has no obvious issues with ears or hearing, to prevent problems.*<br>*Final Visit: Once the tubes fall out, your child should return after 6-12 months so your ear, nose, and throat doctor or other health care provider can check the ears to make sure they are healthy."*<br>**Question Asked on ChatGPT:**<br>When does my child need to be seen again after the tubes are placed?<br>**ChatGPT Answer:**

After the placement of ear tubes (tympanostomy tubes), your child will typically need to be seen for follow-up visits with their healthcare provider. The specific timing of these visits may vary depending on the individual case and the recommendations of the healthcare provider. However, here are some general guidelines:

- Initial Post-operative Visit: An initial post-operative visit is usually scheduled within the first 1 to 2 weeks after the tube placement surgery. During this visit, the healthcare provider will examine the ears, assess the healing process, and check the position and function of the tubes.
- Follow-up Visits: The frequency of subsequent follow-up visits will depend on various factors such as the child's condition, the type of tubes used, and any ongoing concerns. In general, follow-up visits are commonly scheduled every 3 to 6 months, although this can vary.

During these follow-up visits, the healthcare provider will examine the ears, evaluate the tubes' function, and assess the child's overall ear health. They will check for any signs of infection, fluid accumulation, or other issues related to the middle ear. The follow-up visits provide an opportunity to monitor the effectiveness of the tubes and make any necessary adjustments to the treatment plan.

It's important to attend these follow-up visits as scheduled and to communicate any changes in your child's symptoms, such as recurrent ear infections, hearing difficulties, or other concerns. The healthcare provider will determine the appropriate timing for subsequent visits based on your child's progress and specific needs.

It's worth noting that the follow-up schedule may vary for each child, and the healthcare provider may adjust it based on the individual's condition and response to treatment. Therefore, it's best to consult with the healthcare provider who performed the tube placement surgery for specific recommendations regarding the timing and frequency of follow-up visits for your child.

Assessment of ChatGPT's Response:

- Closely aligned with the guidelines
- Communicated the importance of seeking a clinician's expertise

---

Statement 21: Complications associated with ear tubes

AAO-HNSF Guide-lines*:*

"Scarring. A white mark from scarring (sclerosis) or a small depression or pocket may be seen on the eardrum, but this usually does not affect hearing or cause infections and is usually of no concern.

- *Perforation. About 1-2 out of every 100 children will still have a hole (perforation) in the eardrum after a short-term tube falls out, with up to in every 5 children having a perforation after a long-term tube. The hole will often close on its own, but if it does not, it can be repaired in the operating room as a day surgery procedure.*
- *Tubes falling in. Tubes almost always fall out of the ear drum into the ear canal. Very rarely a tube can fall into the middle ear, but usually does not cause any problem and can be removed, if needed.*
- *Tubes not coming out. Most tubes come out within 12 to 24 months. If the tube is still in after 2 to 3 years, or longer, it can be removed.*
- *Tube coming out too early. In rare cases the tube may fall out before 6 months, but many children will have improved by that time. For those who continue to get ear fluid or frequent ear infections a tube may need to be replaced."*

Question Asked on ChatGPT:

What are the possible complications or problems, or ear tubes?

ChatGPT Answer:

While ear tubes (tympanostomy tubes) are generally safe and effective, there can be some potential complications or problems associated with their use. It's important to note that

these complications are relatively rare, and most children experience few or no issues. However, here are some possible complications that can occur:

- Blockage or Obstruction: In some cases, the tubes may become blocked or obstructed by earwax, debris, or tissue. This can impede the drainage of fluid from the middle ear and may require cleaning or removal of the blockage.
- Persistent Otorrhea: Otorrhea refers to drainage or discharge from the ear. While it's normal to have some drainage immediately after tube placement, persistent or prolonged otorrhea can occur. It may be due to infection or inflammation and might require treatment with antibiotic ear drops or other interventions.
- Tube Extrusion: Ear tubes are designed to eventually fall out or extrude from the eardrum as part of the natural healing process. However, in some cases, the tubes can extrude too early or too late. Early extrusion can lead to recurrent ear infections or fluid accumulation, while delayed extrusion may require surgical removal.
- Tympanosclerosis: Tympanosclerosis is the formation of small, white patches of calcium deposits on the eardrum or the tube itself. It usually doesn't cause any symptoms but can occasionally affect hearing. Severe cases may require further treatment.
- Perforation: In rare instances, the eardrum can develop a small hole or perforation during or after tube placement. This can occur due to trauma, infection, or improper tube insertion. Most perforations heal on their own, but larger or persistent perforations may require additional treatment.
- Scarring or Adhesions: Scar tissue or adhesions can occasionally form on the eardrum after tube placement. These can affect hearing or cause other problems. In some cases, surgical intervention may be needed to address the scarring or adhesions.
- Persistent Middle Ear Infections: While ear tubes are intended to reduce the frequency of middle ear infections, it's possible for some children to still experience infections de-spite the tubes. If recurrent infections persist, further evaluation and management may be required.

It's important to discuss the potential risks and complications associated with ear tubes with your healthcare provider. They can provide you with specific information based on your child's case and address any concerns or questions you may have. Remember that the vast majority of children experience significant benefits from ear tubes with minimal com-plications.

Assessment of ChatGPT's Response:

- Closely aligned with the guidelines
- Communicated the importance of seeking a clinician's expertise

---

Statement 22: Water exposure

AAO-HNSF Guidelines:

"Your child will not usually need ear plugs for swimming and bathing while the tubes are in place and open. Headbands or other special efforts to keep water from entering the ear canal are also unnecessary, but may be helpful in the following situations:

- *Pain or discomfort when water enters the ear canal*
- *Current fluid or drainage from the ear canal (an ear infection with the tube), or your child has had frequent drainage*
- *Swimming in lakes or non-chlorinated pools that are not clean*
- *Dunking head in the bathtub (soapy water passes through the tiny hole in the tube easier than plain water)*

There are several types of soft ear plugs or ear putty available, as well as neoprene headbands to cover the ears. NEVER use Play-Doh or Silly Putty as an ear plug—these materials can become trapped in the ear canal and even require surgical removal."

Question Asked on ChatGPT:

Does my child need ear plugs when exposed to water?

ChatGPT Answer:

	Does my child need ear plugs when exposed to water?<br>The use of ear plugs or other water precautions for children with ear tubes (tympanostomy tubes) depends on the specific recommendations of the healthcare provider who inserted the tubes. While there is no universal consensus on this matter, here are some general considerations:<br>• Healthcare Provider's Recommendation: It is important to follow the guidance of your child's healthcare provider regarding water precautions. Some providers may recommend using ear plugs or other measures to keep water out of the ears, while others may not find it necessary.<br>• Healing Period: During the immediate healing period after tube placement, your healthcare provider may advise against water exposure to allow the surgical site to heal properly. This may include avoiding swimming or submerging the head in water for a certain period. Once the healing period is over, your provider may provide further guidance on water precautions.<br>• Individual Factors: The decision to use ear plugs may also depend on individual factors such as the specific condition, the type of tubes used, the presence of other ear issues, and the child's risk of water-related complications. For example, if your child has a history of recurrent ear infections triggered by water exposure, your healthcare provider may recommend using ear plugs.<br>• Water Activities: The nature and duration of water activities may also influence the need for water precautions. For instance, swimming in a pool or calm water may pose less risk compared to activities like diving or vigorous water sports that may increase the chance of water entering the ears.<br>It's important to have a discussion with your child's healthcare provider to understand their recommendations regarding water precautions. They can provide specific guidance based on your child's individual situation and advise on the best approach to protect the ears and minimize the risk of complications.<br>Remember that water precautions are typically temporary and may not be necessary for the entire duration that the tubes are in place. Regular follow-up visits with your healthcare provider will allow them to assess the condition of the ears and provide further instructions regarding water exposure as needed.<br>**Assessment of ChatGPT's Response:**<br>• Closely aligned with the guidelines<br>• Communicated the importance of seeking a clinician's expertise
**Statement 23: Calling ENT**	**AAO-HNSF Guide-lines:**<br>• *Your child's regular doctor or health care provider can't see the tube in the ear, or the tube is blocked*<br>• *Your child has a hearing loss, continued ear infections or continued ear pain/discomfort*<br>• *Ear drainage continues for more than 7-10 days without improvement with treatment*<br>• *Drainage from the ear occurs frequently or more than you think should happen*<br>• *There is wax build-up in the ear canal that doesn't allow the tube to be seen."*<br>**Question Asked on ChatGPT:**<br>When should I call the ENT for my children who had a tympanostomy with ear tubes?<br>**ChatGPT Answer:**<br>After your child has undergone a tympanostomy with ear tube placement, it's important to be aware of certain signs or symptoms that may warrant contacting the ENT (ear, nose, and throat) specialist. While the specific guidelines may vary, here are some general situations in which you should consider reaching out to the ENT:<br>• Persistent or Recurrent Ear Infections: If your child continues to experience recurrent ear infections despite the presence of ear tubes, it is important to inform the ENT. This includes symptoms such as ear pain, fever, irritability, or drainage from the ear.

- Persistent or Worsening Hearing Problems: If you notice that your child's hearing difficulties persist or worsen after tube placement, it's advisable to contact the ENT. This can include issues like persistent hearing loss, difficulty understanding speech, or changes in their response to sound.
- Persistent or Prolonged Ear Discharge: While some drainage immediately after tube placement is normal, persistent or prolonged ear discharge (otorrhea) may indicate an infection or other complications. If the discharge persists or increases, contacting the ENT is recommended.
- Unresolved Middle Ear Fluid: If you notice that fluid continues to accumulate in the middle ear despite the presence of ear tubes, it's important to inform the ENT. Ongoing fluid accumulation can affect hearing and increase the risk of complications.
- Behavioral or Developmental Changes: If you observe significant changes in your child's behavior, such as increased irritability, changes in sleep patterns, or developmental regression, it may be indicative of an issue related to the ears. Informing the ENT about these changes is important for further evaluation.
- Other Concerns or Questions: If you have any other concerns, questions, or uncertainties about your child's condition, it's always appropriate to contact the ENT for clarification or guidance.

Remember, the ENT specialist who performed the procedure is the best person to address any concerns related to your child's specific case. They can provide personalized advice based on the individual circumstances and guide you on when it is necessary to schedule a follow-up visit or seek further evaluation.

If you are unsure about whether to contact the ENT, it is generally better to err on the side of caution and reach out to them for guidance.

Assessment of ChatGPT's Response:

- Closely aligned with the guidelines
- Communicated the importance of seeking a clinician's expertise

AAO-HNSF guideline*: *American Academy of Otolaryngology–Head and Neck Surgery Foundation "Clinical Practice Guideline: Tympanostomy Tubes in Children (Update)"*
